# Combined Effect of Spent Mushroom Substrate and Agro-Industrial Residues on *Pleurotus columbinus* Production and Intra-Cellular Polysaccharide Synthesis

**DOI:** 10.3390/biotech14020034

**Published:** 2025-05-02

**Authors:** Marianna Dedousi, Chrysavgi Gardeli, Seraphim Papanikolaou, Panagiota Diamantopoulou

**Affiliations:** 1 Laboratory of Edible Fungi, Institute of Technology of Agricultural Products, Hellenic Agricultural Organization—Dimitra, 1 Sof. Venizelou, 14123 Lykovryssi, Greece; mdedousi@aua.gr; 2 Laboratory of Food Chemistry and Analysis, Department of Food Science and Human Nutrition, Agricultural University of Athens, 75 Iera Odos, 11855 Athens, Greece; agardeli@aua.gr; 3 Laboratory of Food Microbiology and Biotechnology, Department of Food Science and Human Nutrition, Agricultural University of Athens, 75 Iera Odos, 11855 Athens, Greece; spapanik@aua.gr

**Keywords:** mushrooms, spent coffee grounds, faba bean residues, pistachio shells, biological efficiency, carbohydrates, laccase

## Abstract

Spent mushroom substrate (SMS), spent coffee grounds from espresso production (SCG), faba bean harvest residues (FBR), pistachio shells (PS) wheat straw (WS) (control) agro-industrial waste were combined in different ratios, with or without supplements (wheat bran, soybean flour), to create novel substrates for *Pleurotus columbinus* growth. The impact of the substrates on the mycelial growth rate (Kr), biomass production, laccase, total cellulases and carbohydrate synthesis, along with the C and N consumption by *P. columbinus*, were examined in fully colonized substrates. The incubation period, earliness and biological efficiency (B.E.) (%) were also determined. Then, the intracellular polysaccharide (ICP) contents of the *P. columbinus* produced mushrooms were evaluated in the most promising substrates. *P. columbinus* was grown successfully in a wide range of C/N ratios of substrates and the fastest Kr (7.6 mm/d) was detected on the 70 SMS-30 FBR, without supplements, whereas substrates consisting of SCG enhanced biomass production (700.0–803.7 mg/g d.w.). SMS and PS or SCG led to the shortest incubation and earliness period of *P. columbinus*. The C content was reduced and the N content was substantially increased in all the colonized substrates. The 70 SMS-30 FBR and 80 SMS considerably enhanced the laccase production (up to 59,933.4 U/g d.w.) and substrates consisting of PS promoted total cellulases activities. Greater amounts of carbohydrates (3.8–17.4 mg/g d.w.) than that in the control were recorded for all the substrates. The combination of SMS and SCG or WS led to the highest B.E. values (59.3–87.1%) and ICP amounts (34.7–45.9%, *w*/*w*), regardless of the supplement addition. These findings support the effective utilization of agro-industrial waste in *P. columbinus* cultivation, producing high-value-added compounds and supporting mushroom growth.

## 1. Introduction

The cultivation of edible mushrooms has been increased in the last years, as people world-wide include mushrooms in their everyday diets [1,2]. In addition to their unique culinary properties, mushrooms are low in calories and fat and rich in protein, including all nine essential amino acids, making them a valuable alternative to meat [3,4]. Along with the increase in global mushroom production, the by-products of the mushroom industry are also increasing. These by-products include misshapen mushrooms, mushroom stems and spent mushroom substrate (SMS), which is the most abundant, as three to five kilograms of this lignocellulosic by-product is generated per kilogram of fresh mushrooms [5]. This by-product is traditionally disposed of through landfilling, causing environmental issues.

The rapid expansion of the food industries and agricultural sector has led to the annual generation of substantial amounts of agro-industrial waste. Coffee is one of the world’s most widespread beverages. Spent coffee grounds (SCG) are the solid residues derived after coffee beverage preparation [6]. Most of these residues are commonly thrown directly in the trash and thus end up in landfills, creating environmental issues due to the significant amounts of toxic organic components (caffeine, tannins, polyphenols) that demand great quantities of oxygen to decompose [6,7]. Furthermore, a remarkable increase in the consumption of legumes has been noticed during the last decade, as they are considered a valuable protein source, especially for vegetarians [8]. Consequently, the production of the post harvesting residues of legumes has increased at the same pace. *Pisum sativum* L., included in the *Fabaceae* family, is the second most important crop after the common bean (*Phaseolus vulgaris* L.) and is cultivated in many countries, including Greece [9,10]. Inappropriate management of the post harvesting residues of legumes, like burning, leads to air pollution. Another agricultural by-product produced considerably in the Mediterranean region is pistachio shells (PS), derived from *Pistacia vera* L. cultivation. This waste is mainly subjected to dumping and burning without any treatment [11].

An environmentally friendly way to handle all the above lignocellulosic residues is their reutilization as substrates in mushroom cultivation through solid-state fermentation (SSF). In most cases, the final mixtures of these residues are supplemented with carbon- and nitrogen-rich materials, such as wheat bran and soybean flour. However, substrates without supplements have also been tested and, in many cases, their absence did not result in a negative effect on fungal growth [12,13,14]. This constitutes an economical, viable method of substrate preparation. Oyster mushrooms (those of the genus *Pleurotus*) especially are able to colonize and grow on a wide range of agro-industrial residues, owing to their complex enzymatic system [15]. Ligninolytic enzymes, such as laccase, one of the oxidative enzymes that is secreted in large amounts by oyster mushrooms, and hydrolytic enzymes, like cellulases, have many applications in the food and beverage industry [16,17,18]. Furthermore, during SSF, many bioactive compounds are synthesized by mushrooms, such as N-acetyl-D-glucosamine, which constitutes the structural polysaccharide of the fungal cell wall and carbohydrates, which appear to have exceptional medicinal properties [19].

*Pleurotus columbinus*, commonly known as blue oyster mushroom, is well known for its attractive color and savory flavor, which is almost poultry-like, yet it has not been examined to a great extent for its nutritional value and ability to colonize many different substrates (lignocellulosic residues and agro-industrial wastes). Other researchers have cultivated *P. columbinus* on various residues, such as *Cynodon dactylon* grass weed, African mahogany tree leaves, faba bean straw, rice straw, wheat straw, sugarcane bagasse [20], chopped office papers, cardboard, sawdust and plant fibers [21]. To the best of our knowledge, no other research has been conducted on evaluating SMS, alone or combined with agro-industrial residues, as a cultivation substrate for *P. columbinus* SSF. Generally, only a few studies have examined the suitability of non-supplemented substrates in oyster mushrooms. Furthermore, *P. columbinus* is suggested as a healthy food. It is a rich source of protein and high amounts of vitamin C and vitamin D have been detected in its extracts [22,23]. Moreover, *P. columbinus* has high cellulolytic activities [24] and many researchers have mentioned its high antioxidant and antimicrobial activities, which offer potential applications in the formulation of food or medicinal supplements [22,25].

Thus, the objective of the present study was to evaluate the suitability of novel cultivation substrates consisting of SMS, alone or combined with agro-industrial residues, with or without supplements, in the growth of *P. columbinus*, a mushroom that has not been largely examined. A multifactor experimental design was used including numerous substrate compositions of SMS, SCG, fava bean harvest residues (FBR), PS and wheat straw (WS). The biomass, laccase and carbohydrate production were assessed in fully colonized substrates, along with the remaining carbon and nitrogen amounts. Initially, the selection of the most promising substrates for experiments in bags was based on the results of the *P. columbinus* growth rate conducted in a Petri dish. Then, essential parameters of the growth and fructification (incubation and earliness duration, biological efficiency) were determined, as well as the intra-cellular polysaccharide (ICP) contents in the *P. columbinus*-produced mushrooms.

## 2. Materials and Methods

### 2.1. Strain and Spawn Preparation

*Pleurotus columbinus* (strain AMRL 198), maintained on Potato Dextrose Agar (PDA) (Merck, Darmstadt, Germany), was obtained from the fungal culture collection of the Laboratory of Edible Fungi/Institute of Technology of Agricultural Products/Hellenic Agricultural Organization—Dimitra. Grain spawn of *P. columbinus* was prepared as previously described by Philippoussis et al. [26].

### 2.2. Substrates and Fermentation Parameters

All residues originated from Greek farms and industries. Spent mushroom substrate (SMS) was derived from the large-scale cultivation of *Pleurotus ostreatus* mushrooms conducted by the agricultural industry, Manitus S.A., Paiania, Attica. Faba bean harvest residues (FBR) were obtained after the cultivation of *Pisum sativum* L. by the agricultural industry Okto Adelphia, on the Aegean island Schinoussa. Pistachio shells (PS) came from pistachio nut trees, *Pistacia vera* L., cultivated in the region of Megara, Attica. The industrial residue, spent coffee grounds from espresso production (SCG), was derived from a local coffee shop of Lycovryssi, Attica. Wheat straw (WS), the main commercial cultivation substrate for *Pleurotus* mushrooms, was used as the control substrate and in combination with SMS. All the residues were subjected to chemical analyses for their organic matter [27], total nitrogen and protein contents [28] and cellulose and lignin contents [29,30,31]. All samples were analyzed in triplicate.

Substrate formulations were prepared (on a dry substrate weight basis) by supplementing SMS and each residue with wheat bran (WB) and soybean flour (SF) (40:40:15:5; 60:20:15:5). Also, SMS and WS (control) with supplements (80:15:5) were tested. Otherwise, SMS was mixed with SCG, FBR, PS and WS (70:30, 50:50) and formed new substrates without any supplement, whereas one substrate consisted only of SMS (100 SMS). To prepare the substrates, WS and FBR were mechanically cut to 2–5 cm (cutting machine: Novital, mod. Magnum-4 V, Novelty, Palermo, Italy) and PS were milled to < 0.2 mm (analytical mill Janke & Kunkel, IKA-WERK, Staufen im Breisgau, Germany). After soaking in a specific quantity of tap water for ~12 h to achieve the optimum moisture content (60–75%), some substrates were supplemented with WB and SF (supplemented substrates), while others were utilized without supplements. An amount of 1% *w*/*w* calcium carbonate (CaCO_3_; SDS, Peypin, Bouches-du-Rhône, France) was added to all the substrates to obtain a pH value around 7.

Five glass Petri dishes (90 mm) were filled with each substrate and sterilized twice at T = 121 ± 1 °C (1 h, 1.1 atm). They were allowed to cool before they were aseptically inoculated with one agar plug (9 mm) cut from a *P. columbinus* colony grown previously on a PDA (Merck, Darmstadt, Germany) Petri dish at 26 ± 0.5 °C. Then, they were incubated in a chamber (DRAWELL, mod. DW-LBI-400, Shanghai, China) at T = 26 ± 0.5 °C in the dark until the full colonization of the substrate. Petri dishes were used to investigate the ability of *P. columbinus* to colonize the different substrates via the evaluation of the mycelial growth rate (Kr) (mm/d) by measuring the visible penetration of mycelia into the substrate in two perpendicular directions every few days [26]. Based on this screening, three replicates of polypropylene-autoclavable bags (200 g) of each selected substrate were filled, sterilized twice (T = 121 ± 1 °C, 1 h, 1.1 atm) and then inoculated with 3–5% *w*/*w* (on fresh weight basis) *P. columbinus* grain spawn under aseptic conditions. Substrate colonization took place in a growth chamber (ENTERLAB, mod. GROW-1300 h, Terrassa, Spain) at T = 26 ± 0.5 °C and 85% relative air humidity in the dark until full colonization. After that, the conditions were adjusted for the fructification process (T = 18 ± 1 °C, RH: 90%, under cool white light provided by fluorescent lamps for 12 h/day; air exchange rates were controlled to maintain CO_2_ level < 1000 ppm). Two flushes of mushrooms were harvested. The bags were used to examine the incubation and earliness period (elapsed time between the day of substrate inoculation and the day of the first harvest), as well as the biological efficiency (BE%) (the ratio of the weight of fresh mushrooms per d.w. of substrate). Afterwards, the colonized substrates and harvested mushrooms were subjected to several analyses after they were frozen (T = −20 ± 1 °C), dried by a Heto LyoLab 3000 freeze-dryer (Heto-Holten Als, Lillerod, Denmark), milled and sieved.

### 2.3. Analytical Methods

The remaining carbon [27] and nitrogen (Total Kjeldahl Nitrogen Method) [28] contents were determined in the fully colonized substrates. Furthermore, for the extraction of carbohydrates produced by *P. columbinus* on substrates, 2.88 mL distilled water was added to 2 g of fresh fermented substrate at T = 60 °C for 15 min [32]. Carbohydrates were measured according to Diamantopoulou et al. [33] and their concentration was determined by phenol–sulfuric acid assay [34], using glucose as the standard and expressed as equivalent of glucose.

For the laccase (EC 1.10.3.2) and total cellulases extraction, 20 mL phosphate buffer (0.05 M, pH = 5.0; Merck, Darmstadt, Germany) was mixed with 2 g of fresh fermented substrate in 100 mL Erlenmeyer flasks, which were agitated (T = 20–22 °C, 100 ± 5 rpm, 1 h) in a rotary shaker (ZHICHENG ZHWY 211B, Shanghai, China). After vacuum filtration (Whatman No. 2 paper filter, Whatman plc, Kent, UK) to remove the residual particles, the crude extracts were centrifuged at 10,500 rpm for 15 min at T = 4.0 ± 0.1 °C (Micro 22R, Hettich, Kirchlengern, Germany). The supernatants were kept at T = −20 ± 1 °C.

The laccase activity was measured spectrophotometrically with syringaldazine (4-hydroxy-3.5-dime-thoxybenzaldehydeazine; Sigma, Darmstadt, Germany) as the substrate [13]. The absorbance was measured spectrophotometrically at 525 nm (ε = 6.5 × 104 M^−1^ cm^−1^) (Jasco V-530 UV/VIS spectrophotometer, Tokyo, Japan). One unit of laccase is defined as the quantity of enzymes necessary to induce a change in absorbance of 0.001 per minute under the test conditions employed. The laccase activity was expressed in U/g of substrate.

The total cellulases activity was assayed according to the filter paper assay (FPase) method [35]. Briefly, the release of reducing sugars [36] was measured (spectrophotometrically at 540 nm, Jasco V-530 UV/VIS spectrophotometer, Tokyo, Japan) in a reaction mixture containing Whatman 50 mg No. 1 filter paper (1 × 6 cm strip, Whatman plc, Kent, UK) as the substrate in sodium citrate buffer (0.05 M, pH 4.8; Merck, Darmstadt, Germany) at 50 °C after 60 min and T = 50 ± 1 °C. The total cellulases activities were expressed in U/g of substrate.

The quantity of the mycelial mass produced was estimated indirectly using the N-acetylglucosamine (Sigma-Aldrich, Taufkirchen, Germany) method, formed from fungal chitin hydrolysis, as described by Economou et al. [13]. Glucosamine standard curves were obtained using various concentrations of N-acetylglucosamine. The glucosamine concentration was quantified spectrophotometrically at 650 nm (Jasco V-530 UV/VIS spectrophotometer, Tokyo, Japan) and the results were expressed as mg of biomass per g of dry substrate.

Finally, the intra-cellular polysaccharides (ICP) of mushrooms produced on substrates with statistically higher B.E. values than that of the control were quantified according to Diamantopoulou et al. [33] and Liang et al. [37]. In particular, 20 mL of 2.5 M HCl was used to hydrolyze 0.1 g of dried, powdered mushrooms at 100 °C for 20 min. The mixtures were neutralized to a pH of 7 using 2.5 M NaOH. Samples were filtered (through No. 2 Whatman filters, Whatman plc, Kent, UK) and the 3.5-dinitro-2-hydroxybenzoic acid (DNS) assay was applied to determine their ICP (expressed as glucose equivalents), measuring the absorbance at 540 nm [36] (Jasco V-530 UV/VIS spectrophotometer, Tokyo, Japan). All samples were analyzed in triplicate.

### 2.4. Statistical Analysis

Results are presented as the mean ± standard deviation from a minimum of three replicates. Analysis of variance was followed by Duncan’s *t*-test at the 5% level of probability for assessing differences between means (Statgraphics Centurion XVII, version 17). Pearson’s correlation coefficient was used for determining the relationships (at significance levels of 0.05) between the variables.

## 3. Results and Discussion

### 3.1. Raw Materials, Substrate Analysis and Mycelium Growth Rate

To prepare the final mixtures of SMS and raw agro-industrial residues, their basic chemical properties were determined (Table 1). SCG presented the highest amount of C, N and protein (except for the supplements WB and SF). As shown in Table 2, there was a variety of C/N ratios of the final supplemented cultivation substrates (22.7–34.0% d.w.), whereas higher ratios in non-supplemented cultivation substrates were calculated in Table 3 (32.0–54.8% d.w.). These values are within the acceptable limits, as they support the growth of most mushroom species [26,38]. In a previous study, *P. ostreatus* was cultivated on substrates consisting of WS, SCG and olive pruning residues in different ratios and the C/N ratio ranged from 26 to 69 [39]. High cellulose and lignin contents were observed in substates consisting of WS and PS, respectively, regardless of the supplement addition.

**Figure 1:**
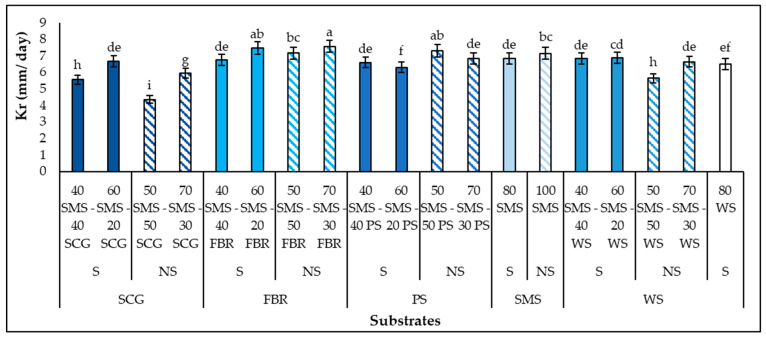
Growth rates (Kr) (mm/d) of *P. columbinus* during solid-state fermentation in glass Petri dishes on substrates consisting of spent mushroom substrate (SMS), spent coffee grounds (SCG), faba bean residues (FBR), pistachio shells (PS) and wheat straw (WS), supplemented (S) or non-supplemented (NS). Values are expressed as means ± standard errors of means. Lack of letters in common indicates statistically significant differences (Duncan’s *t*-test, <0.05) among different substrates.

Firstly (Figure 1), the effect of all the different substrates on *P. columbinus* growth was evaluated by examining its mycelium growth rate (Kr) (mm/d). *P. columbinus* grew successfully in all the substrates and the combination of SMS and FBR, without supplements 70 SMS-30 FBR led to the fastest Kr (7.6 mm/d). Among the highest Kr values were also detected on the 60 SMS-20 FBR (7.5 mm/d) and 50 SMS-50 PS (7.3 mm/d). In contrast, Philippoussis et al. [26] have mentioned the negative impact of PS substrates on *Pleurotus* strain growth. It is worth mentioning that the absence of additives in the final substrates did not negatively affect the Kr values. This has also been observed in *Pleurotus citrinopileatus* and *P. ostreatus* grown on different substrates with SMS and the hydroponic roots of leafy vegetables without supplements [12,40]. Additionally, Economou et al. [13] reported that the highest *P. ostreatus* and *Pleurotus pulmonarius* Kr values were observed in SMSs, without supplements, while the supplementation of SMS with WB and SF led to a decrease in the Kr. The slowest Kr values were recorded when *P. columbinus* was cultivated on 50 SMS-50 SCG (4.4 mm/d). The Kr values and C concentrations of the cultivation substrates were negatively and significantly related (r^2^ = −0.5368). In any case, the Kr values of this study are among the highest of those reported in the literature for *Pleurotus* cultivation on SMSs (1.76–8.60 mm/d) [13,41,42], indicating the suitability of these combinations, SMSs with agro-industrial residues, as cultivation mushroom substrates. Therefore, for the subsequent experiments (cultivation in polypropylene bags), ten substrates combining SMS and each agro-industrial residue with (and without) supplements were selected based on the results of the fastest Kr values (Table 4).

### 3.2. Analysis of Substrates After *P. columbinus* Colonization

All the selected substrates consisting of SMS and agro-industrial residues successfully supported the production of *P. columbinus* mushrooms during the experiment in small polypropylene bags. The incubation period lasted from 9 to 16 days (Table 4). It is important to mention that *P. columbinus* needed less time to fully colonize all the new, alternative substrates than the control one, while the substrates consisting of SMS and PS (40 SMS-40 PS, 50 SMS-50 PS) led to the shortest incubation period. Otherwise, the substrate composition did not influence the colonization period of *P. ostreatus* (7–9 days) [39,43]. Short cultivation periods in the mushroom industry are preferable to minimize the high risk of contamination [38]. In a previous study, *P. columbinus* colonized chopped office papers and cardboard within two weeks after spawning, similar to this study, whereas more time, up to four weeks, was needed in sawdust and plant fibers [21]. Also, a strong negative correlation was recorded for the incubation period and lignin concentration of the cultivation substrates (r^2^ = −0.941).

The differences in the biomass (279.2 to 803.7 mg/g d.w.) among the colonized substrates were statistically significant (Table 4). In particular, the results of the present study indicate the positive effect of SCG addition in biomass production. The greatest biomass production was observed in the non-supplemented substrates 70 SMS-30 SCG and in 60 SMS-20 SCG, biomass production was great enough (700.0 mg/g d.w.). Diamantis et al. [44] have also reported that the addition of coffee residue in WS and beech wood shavings led to a significant increase in the biomass production by *P. ostreatus*. Furthermore, the negative correlation between the growth rate and biomass production have been reported [45]. In the present study, the greatest biomass production (803.7 mg/g d.w.) and the lowest Kr (5.9 mm/d) were detected in the 70 SMS-30 SCG. In a previous study, significantly lower biomass (104.90–351.88 mg/g d.w.) was produced by *P. ostreatus* and *P. eryngii* cultivated on substrates consisting of SMS and agro-industrial residues [41].

The carbon (C_rem_, %) and nitrogen (N_rem_, %) contents were also measured in the substrates after they were fully colonized by *P. columbinus* (Table 4). The C_rem_ contents of these samples were reduced, while the highest reduction in the C_rem_ was observed for the 60 SMS-20 WS. The decrease in C can be explained by the breakdown of organic matter in the substrate. Otherwise, the N_rem_ contents of the fully colonized substrates were substantially increased. The greatest change in the N_rem_ contents was detected for the 70 SMS-30 FBR. The increase in the N contents in substrates after colonization or harvest has already been mentioned in previous studies [46,47,48]. This increase may be related to the high protein content of the heavily grown mycelium in the substrate, or because fungi secrete proteinaceous extracellular enzymes by degrading the by-products during fermentation, which are metabolized [49]. As was expected, the initial C and N amounts on the cultivation substrates were highly positively correlated with the amounts of the C_rem_ and N_rem_ (r^2^ = 0.7252 and r^2^ = 0.9051, respectively). Also, the N_rem_ was strong negatively correlated with the substrate C/N and cellulose (r^2^ = −0.8968 and −0.8926, respectively).

### 3.3. Laccase and Total Cellulases Production

**Figure 2:**
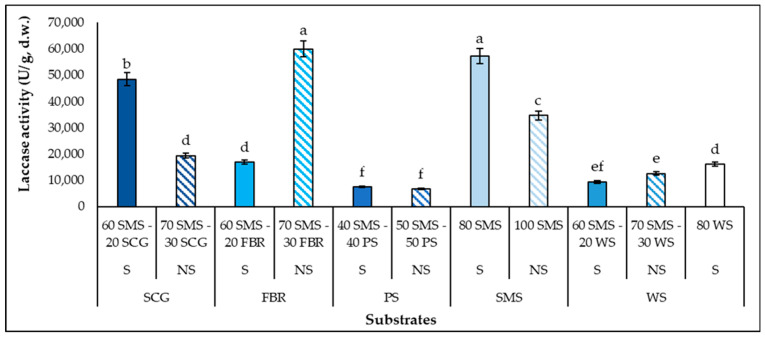
Laccase production (U/g substrate, d.w.) by *P. columbinus* in polypropylene bags on substrates consisting of spent mushroom substrate (SMS), spent coffee grounds (SCG), faba bean residues (FBR), pistachio shells (PS) and wheat straw (WS), supplemented (S) or non-supplemented (NS). Values are expressed as means ± standard errors of means. Lack of letters in common indicates statistically significant differences (Duncan’s *t*-test, <0.05) among different substrates.

Laccase activity is influenced by many factors, including the stage of SSF, the cultivation substrate, the fungal species and the availability of nutrient sources and their concentration [13,50]. In the present study, SMSs, supplemented (80 SMS, 60 SMS-20 SCG) or not (70 SMS-30 FBR, 100 SMS), improved the laccase production (34,646.4–59,933.4 U/g d.w.) by *P. columbinus* (Figure 2) with statistically significant differences from the control substrate (16,013.4 U/g d.w.), while the combination of SMS and PS led to the lowest laccase production. According to the literature, several additives have been reported to enhance laccase production in mushroom cultivation substrates [13,51,52]. In most of the cases of the present study, the additive supplementation had a positive impact on the laccase synthesis, but greater laccase activity was detected in the 70 SMS-30 FBR (non-supplemented) rather than in the 60 SMS-20 FBR (supplemented), indicating that laccase activity is influenced by a combination of factors. In a previous study, the maximum value of laccase enzyme activity was observed in the middle of commercial SMS colonization by *P. pulmonarius* (51,353 U/g d.w.), similar to the greatest laccase activity of the present study, while the greatest laccase activity for *P. ostreatus* was detected at the end of commercial SMS colonization (12,360 U/g d.w.) [53]. Dedousi et al. [41] also reported high laccase production, 62,539.03 U/g d.w., by *P. ostreatus* cultivated on commercial SMS at the end of the cultivation period. Additionally, *P. columbinus* presented the highest laccase activity in different media among other basidiomycetes [24]. It is noteworthy that the laccase activity was negatively correlated with the C_rem_ (r^2^ = −0.6636).

**Figure 3:**
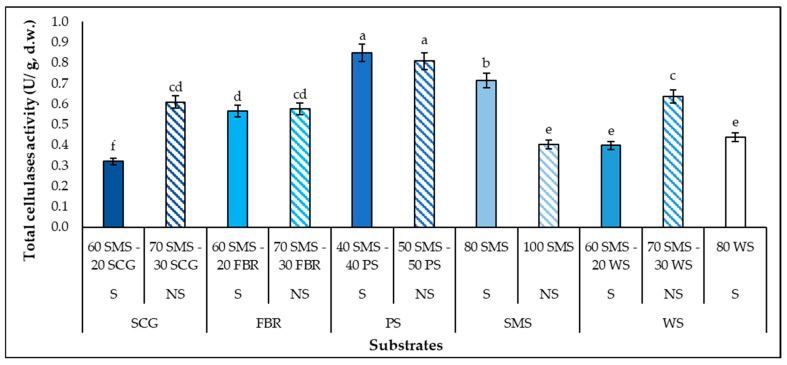
Total cellulases production (U/g substrate, d.w.) by *P. columbinus* in polypropylene bags on substrates consisting of spent mushroom substrate (SMS), spent coffee grounds (SCG), faba bean residues (FBR), pistachio shells (PS) and wheat straw (WS), supplemented (S) or non-supplemented (NS). Values are expressed as means ± standard errors of means. Lack of letters in common indicates statistically significant differences (Duncan’s *t*-test, <0.05) among different substrates.

The total cellulases activity ranged from 0.3 to 0.8 U/g d.w. among the substrates (Figure 3). All the substrates consisting of SMS (except for 60 SMS–20 SCG) enhanced the total celulases production by *P. columbinus*. The highest activity was recorded in 40 SMS–40 PS, while 50 SMS–50 PS also enhanced total cellulases production. On the contrary, it is important to mention that substrates consisting of PS had a negative effect on the laccase production. Khalil et al. [54] reported that, cultivated on sawdust, sugarcane bagasse and paddy straw, *P. ostreatus* produced 0.86–3.51 U/g total cellulases and *Pleurotus sajor-caju* produced 0.19–0.83 U/g, values similar to those of the present study. Higher total celulases production was recorded for *P. ostreatus* cultivated on wheat straw, tree leaves and apple and banana peels (1.6–3.4 U/mL) [55] than that of the present study. Whilst the ability of *P. columbinus* to produce total cellulases and generally hydrolytic enzymes has not been widely studied yet, *Pleurotus* spp. have been tested for their ability to produce not only total cellulases but also endoglucanase (EC 3.2.1.4) [41,56], exoglucanase (EC 3.2.1.91) [54,56] and β-glucosidase (EC 3.2.1.21) [56,57].

Many researchers have studied the synthesis of enzymes, as they have multiple applications in several industries, including food and dairy production. Although enzymes can be isolated from bacteria, plants and animals, their fungal origin is preferable due to the easy preparation and efficient production using inexpensive media.

### 3.4. Carbohydrate Production

**Figure 4:**
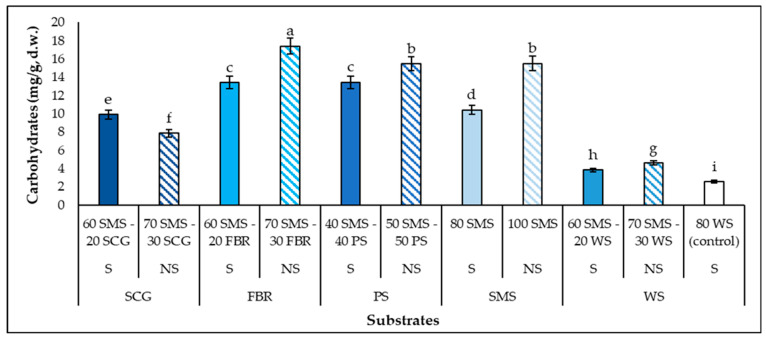
Carbohydrate production (mg/g substrate, d.w.) by *P. columbinus* in polypropylene bags on substrates consisting of spent mushroom substrate (SMS), spent coffee grounds (SCGs), faba bean residues (FBR), pistachio shells (PSs) and wheat straw (WS), supplemented (S) or non-supplemented (NS). Values are expressed as means ± standard errors of means. Lack of letters in common indicates statistically significant differences (Duncan’s *t*-test, < 0.05) among different substrates.

Notable concentrations of carbohydrates were also produced during the *P. columbinus* growth in the different substrates containing SMS. The growth medium and the conditions of the culture and the fungi, both in submerged cultures or SSF, are the most crucial parameters for carbohydrate production [58,59]. Specifically, the carbohydrate concentration was negatively correlated with the substrates’ C (r^2^ = −0.6118), while a high positive correlation was detected with the Kr (r^2^ = 0.6437). According to Figure 4, statistically significant differences were noticed in the production of carbohydrates among the substrates. The SMS had a positive impact on the production of carbohydrates, as greater amounts of them were produced in all the SMS substrates than that of the control (2.6 mg/g d.w.). The highest amount was produced in the 70 SMS-30 FBR (17.4 mg/g d.w.). In general, the enhancement in the carbohydrate production in the non-supplemented substrates was greater than that in the corresponding supplemented ones. In a previous study, Economou et al. [53] reported significantly lower exopolysaccharide production by *P. ostreatus* (2.40 mg/g d.w.) and *P. pulmonarius* (3.18 mg/g d.w.) cultivated on SMS at the end of colonization. Similarly, Melanouri et al. [58] reported even lower exopolysaccharide production by *P. ostreatus* (0.73–1.82 mg/g d.w.), while greater amounts were produced by *Ganoderma resinaceum* (2.47–5.38 mg/g) and *Lentinula edodes* (1.64–5.68 mg/g). A much higher quantity of extracellular polysaccharides (33.32 g/L) was produced by *P. sajor caju* in submerged fermentation [60]. *P. columbinus* has not so far been studied regarding its ability to produce carbohydrates in liquid cultures or SSF, so the above results are of great interest. Since the fully colonized substrates by *P. columbinus* obtained considerable amounts of carbohydrates, the extraction of these bioactive compounds is really promising for their use in functional food or as valuable compounds for biotechnological applications with remarkable medicinal properties [61,62].

### 3.5. Evaluation of Substrates for *P. columbinus* Cultivation

All substrates had a positive impact on reducing the earliness period (Table 5). *P. columbinus* needed less time to complete the earliness period when it was cultivated on substrates consisting of SMS and SCG or PS, regardless of supplement presence or whether more time was needed on substrates consisting of WS. In the present study, the earliness period was highly positively correlated with the substrate cellulose (r^2^ = 0.6369), whereas there was a strong negative correlation between the earliness period and substrate lignin (r^2^ = −0.7499). Mohamed et al. [20] reported that 19–22 days lapsed to the visible pinhead formation of *P. columbinus* grown on different substrates consisting of FBR, whereas a much longer earliness period (45.67–140.25 days) was needed for *P. columbinus* cultivated on substrates consisting of rice straw supplemented with peels of prickly pear and rice husk [63].

Satisfactory B.E. values were detected in most of the tested substrates (Table 5). The B.E. values (59.3–87.1%) recorded for the substrates consisting of SMS and SCG or WS, regardless of the supplement addition, were equal to or higher than that of the control substrate (61.6%). Also, a high B.E. value was presented in the supplemented substrate of 60 SMS-20 FBR (69.6%), whereas the combination of SMS and FBR without supplements led to the lowest B.E. (23.4%). Contrarily, the 50 SMS-50 PS (without supplements) supported a higher B.E. (57.1%) than the supplemented 40 SMS-40 PS (28.5%). The results of this study reveal that *P. columbinus* can be successfully cultivated on SMS, alone or in combination with other agro-industrial residues and supplemented or not, which is a completely new finding. So, apart from substrate supplementation, there are other crucial physicochemical parameters which can impact the mushroom B.E., such as the availability of nutrients, the cellulose and lignin contents, the pH, the moisture content and the combination of all these factors on the cultivation substrate. It was observed that the B.E. values were very highly positively correlated with the C_rem_ (r^2^ = 0.7092). In addition, the mixtures of SMS and agro-industrial residues (except for 70 SMS-30 FBR and 40 SMS-40 PS) positively influenced the B.E. values, as they were higher than that in the substrate consisting of only SMS (supplemented 80 SMS, 49.7% and non-supplemented 100 SMS, 34.6%).

Mandeel et al. [21] reported similar B.E. values for *P. columbinus* grown on plant fibers and sawdust (66.4–87.7%), while the values (100.8–134.5%) on chopped office papers and cardboard were higher than those of the present study. Also, a wide range of B.E. values was recorded (43.3–99.3%) when *P. columbinus* was cultivated on rice straw and corn straw supplemented with different ratios of compost [64]. On the contrary, substantially low B.E. values were recorded (9.3–14.2%) when *P. columbinus* was cultivated on different substrates consisting of faba bean (*Vicia faba* L.) straw [20]. Moreover, it is noteworthy that the cultivation of *P. columbinus* on substrates consisting of SMS has not yet been examined in other studies. Nevertheless, mixtures of SMS and agro-industrial residues (WS, barley and oat straw, coffee residue, the roots of leafy vegetables) have been tested as alternative substrates in other *Pleurotus* sp. (*P. ostreatus*, *P. eryngii*, *P. citrinopileatus*) cultivations and positively affected the B.E. values [12,41,42,65].

### 3.6. Intra-Cellullar Polysaccharide (ICP) Synthesis in Mushrooms

**Figure 5:**
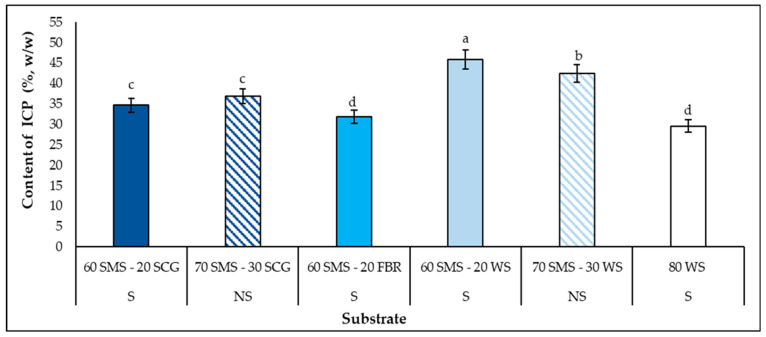
Intra-cellular polysaccharide (ICP) (%, *w*/*w*) synthesis by *P. columbinus* in mushrooms produced during solid-state fermentation in polypropylene bags on selected substrates consisting of spent mushroom substrate (SMS), spent coffee grounds (SCG), faba bean residues (FBR) and wheat straw (WS), supplemented (S) or non-supplemented (NS). Values are expressed as means ± standard errors of means. Lack of letters in common indicates statistically significant differences (Duncan’s *t*-test, <0.05) among different substrates.

Intra-cellular polysaccharides (ICP) were measured in selected mushrooms, those produced on substrates with statistically higher B.E. values than that of the control substrate. As shown in Figure 5, all the substrates consisting of SMS had a positive impact on the ICP synthesis by *P. columbinus.* The highest amount was detected in mushrooms produced on SMS combined with WS, with (45.9%, *w*/*w*) or without supplements (42.5%, *w/w*). The positive effect of WS on the ICP content and generally in mushroom dietary compositions has also been detected in previous studies [39,41], indicating the reason why it is the major substrate of commercial *Pleurotus* sp. cultivation. Moreover, significant ICP contents were observed in mushrooms produced on SMS combined with SCGs (34.7–36.8%, *w*/*w*). Otherwise, the ICP synthesis did not show a statistically significant difference in the mushrooms cultivated on 60 SMS-20 FBR (31.8%, *w*/*w*) and the control substrate (29.6%, *w*/*w*). Economou et al. [53] reported slightly higher values of ICPs in *P. ostreatus* (42.02%, *w*/*w*) and *P. pulmonarius* (42.94%, *w*/*w*) mushrooms grown on SMSs. Similar ICP values to the present results were observed for *P. ostreatus* mushrooms (29–44%, *w*/*w*) cultivated on substrates of different SMSs and the combination of SMSs and fresh agro-residues, while they were higher for *P. eryngii* mushrooms (42–46%, *w*/*w*) in the same study [41]. Furthermore, comparable results to those of the present study have been reported for *P. ostreatus* mushrooms (32.03–44.92%, *w*/*w*) grown on different substrates with SMS and the roots of leafy vegetables, with or without supplements, whereas the highest ICP synthesis was detected in the 90% SMS (50.93%, *w*/*w*) [40].

The quantity of polysaccharides in mushrooms is an important parameter, connected with many health-beneficial properties. Glucans, the main constituent of mushrooms’ polysaccharides, especially have various biological functions [66,67]. So, the selection of the most suitable substrates for the subsequent large-scale experiments will be based on the B.E. values and ICP contents of *P. columbinus* mushrooms, as the final purpose of this study is the production of glucans and their possible incorporation into food supplements.

## 4. Conclusions

The results of the present study reveal the possible bioconversion of substrates consisting of different agro-industrial residues into useful compounds, such as biomass, laccase and carbohydrates, by *P. columbinus*. All the substrates supported *P. columbinus* growth and the Kr values were up to 7.6 mm/day. Higher biomass (424.0–803.7 mg/g d.w.) and carbohydrate (3.8–17.4 mg/g d.w.) synthesis by *P. columbinus* was detected in all the colonized substrates (except for the biomass synthesis on the supplemented substrates 60 SMS-20 FBR and 60 SMS-20 WS) compared with the control substrate (345.2 and 2.6 mg/g d.w., respectively). Moreover, substrates consisting of SMS or SMS with SCG and FBR favored laccase production (34,646.4–59,933.4 U/g d.w.), while those of PS promoted total cellulases synthesis (0.8 U/g d.w.). Regarding the C_rem_ and N_rem_ in the fully colonized substrates, the former was decreased and the latter was increased in all the cases. Also, it is important to mention that although the substrates of only SMS, with (80 SMS) or without (100 SMS) supplements, had a positive influence on the biomass and laccase production the B.E. of *P. columbinus* on these substrates was low. Otherwise, higher or equal B.E values were recorded in substrates consisting of SMS with SCG or WS and 60 SMS-20 FBR (59.3–87.1%) than that of the control substrate (61.6%), so ICP were determined in the produced mushrooms of these substrates. Apart from the 60 SMS-20 FBR, the rest of the substrates promoted greater ICP synthesis (34.7–45.9%, *w*/*w*) by *P. columbinus* than that of the control substrate (29.6%, *w*/*w*). To conclude, this study widens our knowledge on the suitability of alternative substrates, with or without supplements, which have not been used in the cultivation of *P. columbinus*, a not-so-well-tested mushroom. Finally, further large-scale experiments should be conducted to investigate how the most prominent substrates, 60 SMS-20 SCG, 70 SMS-30 SCG, 60 SMS-20 WS and 70 SMS-30 WS, influence the quality of *P. columbinus*, as well as its nutritional and bioactive compounds, such as glucans.

## Figures and Tables

**Table 1:** Chemical composition of raw agro-industrial residues and supplements. Values (% d.w.) are expressed as means ± standard errors of means.

Parameters (%)	SMS *	SCG	FBR	PS	WS	WB	SF
C	29.0 ± 0.1 e **	32.9 ± 0.0 a	29.7 ± 0.6 d	31.1 ± 0.4 c	31.5 ± 0.2 bc	31.7 ± 0.2 b	31.3 ± 0.1 bc
N	0.8 ± 0.0 d	1.2 ± 0.1 c	0.6 ± 0.0 e	0.5 ± 0.0 e	0.5 ± 0.0 e	1.9 ± 0.1 b	6.6 ± 0.1 a
Protein	5.0 ± 0.2 d	7.5 ± 0.4 c	3.6 ± 0.2 e	3.3 ± 0.2 e	3.0 ± 0.0 e	11.6 ± 0.4 b	41.0 ± 0.6 a
Cellulose	37.1 ± 1.0 b	22.6 ± 0.2 d	38.1 ± 0.0 b	33.3 ± 0.7 c	41.2 ± 1.9 a	10.4 ± 0.0 f	13.9 ± 0.1 e
Lignin	7.0 ± 0.3 c	12.0 ± 0.2 b	7.0 ± 0.2 c	14.8 ± 0.5 a	5.7 ± 0.0 d	3.0 ± 0.1 e	7.6 ± 0.2 c

* SMS: spent mushroom substrate; SCG: spent coffee grounds; FBR: faba bean residues; PS: pistachio shells; WS: wheat straw; WB: wheat bran; SF: soybean flour. ** Lack of letters in common indicates statistically significant differences (Duncan’s *t*-test, <0.05) for comparisons of treatment means among different substrates.

**Table 2:** Chemical composition of final supplemented substrates (before inoculation) utilized for solid-state fermentation. Values are expressed as means ± standard errors of means.

Supplemented Substrates	C (% d.w.)	N (% d.w.)	C/N	Cellulose (%, *w*/*w*)	Lignin (%, *w*/*w*)
40 SMS-40 SCG *	30.5 ± 0.9 b **	1.3 ± 0.1 a	22.7 ± 0.5 e	26.5 ± 0.4 e	9.9 ± 0.6 ab
60 SMS-20 SCG	30.2 ± 0.2 bcde	1.3 ± 0.0 ab	23.8 ± 0.9 de	29.6 ± 0.4 d	8.9 ± 0.6 bc
40 SMS-40 FBR	29.7 ± 0.1 cde	1.1 ± 0.0 bc	26.2 ± 1.0 cd	32.3 ± 1.3 bc	7.8 ± 0.7 cd
60 SMS-20 FBR	29.6 ± 0.1 de	1.2 ± 0.1 bc	25.5 ± 1.2 d	32.5 ± 1.3 bc	7.9 ± 0.7 cd
40 SMS-40 PS	30.3 ± 0.1 bcd	1.1 ± 0.0 cd	28.2 ± 1.1 bc	30.8 ± 0.9 cd	11.0 ± 0.9 a
60 SMS-20 PS	29.9 ± 0.1 bcde	1.0 ± 0.0 cd	29.0 ± 1.2 bc	31.7 ± 0.9 bcd	9.4 ± 0.9 b
80 SMS	29.5 ± 0.1 e	1.0 ± 1.0 cd	28.3 ± 1.5 bc	32.7 ± 0.2 abc	6.8 ± 0.6 de
40 SMS-40 WS	30.4 ± 0.2 bc	1.1 ± 0.1 cd	28.8 ± 1.7 bc	33.9 ± 2.1 ab	7.3 ± 0.5 d
60 SMS-20 WS	29.9 ± 0.2 bcde	1.0 ± 0.0 cd	29.2 ± 0.9 b	33.3 ± 2.1 abc	7.6 ± 0.5 cd
80 WS	31.4 ± 0.1 a	0.9 ± 0.0 d	34.0 ± 1.2 a	35.2 ± 2.1 a	5.7 ± 0.6 e

* SMS: spent mushroom substrate; SCG: spent coffee grounds; FBR: faba bean residues; PS: pistachio shells; WS: wheat straw. ** Lack of letters in common indicates statistically significant differences (Duncan’s *t*-test, <0.05) for comparisons of treatment means among different supplemented substrates.

**Table 3:** Chemical composition of final non-supplemented substrates (before inoculation) utilized for solid-state fermentation. Values are expressed as means ± standard errors of means.

Non-Supplemented Substrates	C (% d.w.)	N (% d.w.)	C/N	Cellulose (%, *w*/*w*)	Lignin (%, *w*/*w*)
50 SMS-50 SCG *	30.4 ± 0.8 b **	1.0 ± 0.1 a	32.0 ± 1.5 e	30.3 ± 0.2 e	9.5 ± 0.4 b
70 SMS-30 SCG	30.1 ± 0.1 bc	0.9 ± 0.0 b	34.5 ± 1.6 e	33.4 ± 0.2 e	8.5 ± 0.4 c
50 SMS-50 FBR	29.2 ± 0.2 de	0.7 ± 0.0 c	41.1 ± 0.7 d	37.6 ± 1.1 bcd	7.0 ± 0.5 d
70 SMS-30 FBR	29.1 ± 0.2 de	0.7 ± 0.0 c	43.8 ± 1.1 d	37.8 ± 1.1 bcd	7.0 ± 0.5 d
50 SMS-50 PS	30.0 ± 0.1 bc	0.6 ± 0.0 d	52.2 ± 1 bc	35.7 ± 0.7 d	10.9 ± 0.7 a
70 SMS-30 PS	29.5 ± 0.2 cde	0.6 ± 0.0 d	49.0 ± 2.2 c	36.6 ± 0.7 cd	9.4 ± 0.7 b
100 SMS	28.9 ± 0.2 e	0.6 ± 0.0 d	48.8 ± 2.4 c	38.1 ± 0.0 bc	7.0 ± 0.2 d
50 SMS-50 WS	30.2 ± 0.1 bc	0.6 ± 0.0 de	54.8 ± 2.8 b	39.6 ± 1.9 ab	6.4 ± 0.2 de
70 SMS-30 WS	29.7 ± 0.1 bcd	0.6 ± 0.0 d	51.4 ± 1.7 bc	39.0 ± 1.9 b	6.6 ± 0.2 d

* SMS: spent mushroom substrate; SCG: spent coffee grounds; FBR: faba bean residues; PS: pistachio shells; WS: wheat straw. ** Lack of letters in common indicates statistically significant differences (Duncan’s *t*-test, <0.05) for comparisons of treatment means among different non-supplemented substrates.

**Table 4:** *P. columbinus* production parameters at the end of colonization in polypropylene bags on final mixtures, supplemented or non-supplemented. Values are expressed as means ± standard errors of means.

	Substrates	Incubation Period (Days)			Biomass (mg/g d.w.)			C_rem_ (%, *w*/*w*)			N_rem_ (%, *w*/*w*)		
Supplemented	60 SMS-20 SCG *	12 ± 0	f **	C ***	700.0 ± 17.2	c	B	29.6 ± 0.9	a	A	1.7 ± 0.1	ab	AB
	60 SMS-20 FBR	13 ± 1	de	B	279.2 ± 12.8	h	D	29.1 ± 0.4	b	B	1.6 ± 0.1	bc	B
	40 SMS-40 PS	10 ± 0	g	D	558.2 ± 17.2	d	C	28.8 ± 0.5	c	C	1.7 ± 0.0	a	A
	80 SMS	14 ± 0	c	A	746.0 ± 21.0	b	A	27.4 ± 0.2	e	D	1.4 ± 0.0	de	C
	60 SMS-20 WS	13 ± 0	d	B	313.9 ± 12.8	gh	D	29.0 ± 0.0	bc	BC	1.3 ± 0.0	ef	C
Non-supplemented	70 SMS-30 SCG	12 ± 1	ef	C	803.7 ± 22.9	a	A	29.1 ± 0.1	b	B	1.5 ± 0.1	cd	A
	70 SMS-30 FBR	16 ± 0	a	A	494.7 ± 12.2	e	C	27.5 ± 0.2	de	C	1.3 ± 0.1	ef	B
	50 SMS-50 PS	9 ± 0	h	D	554.9 ± 17.2	d	B	29.6 ± 1.6	a	A	1.0 ± 0.1	g	C
	100 SMS	16 ± 1	a	A	756.0 ± 24.8	b	A	27.7 ± 0.2	d	C	0.8 ± 0.0	f	D
	70 SMS-30 WS	15 ± 0	b	B	424.0 ± 19.9	f	D	28.9 ± 0.7	bc	B	0.8 ± 0.1	h	D
	80 WS (control)	16 ± 0	a		345.2 ± 14.7	g		29.5 ± 0.3	a		1.3 ± 0.0	h	

* SMS: spent mushroom substrate; SCG: spent coffee grounds; FBR: faba bean residues; PS: pistachio shells; WS: wheat straw. ** Lack of lowercase letters in common indicates statistically significant differences (Duncan’s *t*-test, <0.05) among all substrates. *** Lack of capital letters in common indicates statistically significant differences (Duncan’s *t*-test, <0.05) among supplemented or non-supplemented substrates.

**Table 5:** Earliness period (days) and biological efficiency (B.E.) (%) of *P. columbinus* on supplemented or non-supplemented substrates in polypropylene bags. Values are expressed as means ± standard errors of means.

	Substrates	Earliness (Days)			B.E. (%)		
Supplemented	60 SMS-20 SCG *	19 ± 0	f **	C ***	87.1 ± 1.5	a	A
	60 SMS-20 FBR	21 ± 1	de	B	69.6 ± 2.1	b	B
	40 SMS-40 PS	19 ± 1	f	C	28.5 ± 0.7	h	E
	80 SMS	20 ± 0	ef	BC	49.7 ± 1.9	f	D
	60 SMS-20 WS	24 ± 1	c	A	59.3 ± 2.3	de	C
Non-supplemented	70 SMS-30 SCG	20 ± 1	f	C	63.4 ± 1.2	c	A
	70 SMS-30 FBR	27 ± 1	a	A	23.4 ± 0.6	i	D
	50 SMS-50 PS	19 ± 1	f	C	57.1 ± 1.6	e	B
	100 SMS	22 ± 1	cd	B	34.6 ± 0.6	g	C
	70 SMS-30 WS	25 ± 1	b	A	63.9 ± 1.7	c	A
	80 WS	27 ± 1	ab		61.6 ± 2.4	cd	

* SMS: spent mushroom substrate; SCG: spent coffee grounds; FBR: faba bean residues; PS: pistachio shells; WS: wheat straw. ** Lack of lowercase letters in common indicates statistically significant differences (Duncan’s *t*-test, <0.05) among all substrates. *** Lack of capital letters in common indicates statistically significant differences (Duncan’s *t*-test, <0.05) among supplemented or non-supplemented substrates.

## Data Availability

The original contributions presented in this study are included in the article. Further inquiries can be directed to the corresponding author.
